# Infections in primary sclerosing cholangitis and inflammatory bowel disease: a systematic review and meta-analysis

**DOI:** 10.1093/jcag/gwaf023

**Published:** 2025-09-03

**Authors:** Navneet Natt, Tyrel Jones May, Gurpreet Malhi, Jennifer Dumond, Aliya Gulamhusein, Parul Tandon

**Affiliations:** Division of Gastroenterology, Department of Medicine, Western University, London, Ontario N6A 5C1, Canada; Division of Gastroenterology and Hepatology, University Health Network, University of Toronto, Toronto, Ontario M5T 2S8, Canada; Division of Gastroenterology, Department of Medicine, Western University, London, Ontario N6A 5C1, Canada; Northern Ontario School of Medicine University, Thunder Bay, Ontario P7B 7A5, Canada; Toronto Centre Liver for Disease, University Health Network, University of Toronto, Toronto, Ontario M5T 2S8, Canada; Division of Gastroenterology and Hepatology, University Health Network, University of Toronto, Toronto, Ontario M5T 2S8, Canada

**Keywords:** infections, PSC-IBD, PSC, IBD

## Abstract

**Purpose:**

Primary sclerosing cholangitis (PSC) is a cholestatic liver disease that frequently coexists with inflammatory bowel disease (IBD). The risk of infections in patients with concurrent PSC-IBD remains unclear. The aim of this study was to identify the event rate of infections and associated risk factors in PSC-IBD patients.

**Methods:**

MEDLINE, Embase, and the Cochrane Central Register of Controlled Trials were searched from inception to September 12, 2024 for studies examining event rate or risk factors for infection in patients with PSC-IBD. The primary outcome was the event rate of all-cause and site-specific infections as well as infection-related mortality. The secondary outcome was risk factors for infection. Random-effects models were used to calculate pooled odds ratios (OR) with 95% confidence intervals (CI) comparing the event rate of all-cause infections in PSC-IBD patients to those with just PSC and just IBD. *I*^2^ values more than 50% suggested substantial heterogeneity.

**Results:**

Eighty-one studies were included. The pooled event rate of all-cause infections in patients with PSC-IBD was 25.1% (95% CI, 17.0%-33.2%, *I*^2^ = 99.2%). PSC-IBD patients had significantly increased odds of all-cause infection (OR 3.67, 95% CI, 2.07-6.52, *I*^2^ = 41.9%), sepsis (OR 3.35, 95% CI, 2.29-4.91, *I*^2^ = 9.1%), and infection-related mortality (OR 11.25, 95% CI, 2.03-62.37, *I*^2^ = 0) compared to those with IBD but not those with PSC.

**Conclusion:**

Patients with PSC-IBD appear to be at increased risk of all-cause infection, sepsis, and mortality compared to those with IBD alone.

## Introduction

Primary sclerosing cholangitis (PSC) is an immune-mediated liver disease leading to progressive fibrosis of the intra- and/or extrahepatic bile ducts.^1^ Stricturing of the biliary tree can predispose patients to developing cholangitis, biliary sepsis, and decompensated cirrhosis necessitating liver transplant (LT).^1^^,^^2^ Up to 80% of patients with PSC may develop inflammatory bowel disease (IBD), which includes ulcerative colitis (UC), Crohn’s disease (CD), and IBD unclassified.^2^ Those with concurrent PSC and IBD (PSC-IBD) represent a unique cohort of patients with a distinct disease phenotype and elevated risk of complications.^2^ However, the risk of infection in this group is not clearly defined.

Patients with IBD may have a heightened risk of infection due to underlying immune dysregulation and immunosuppressive therapy.^3^ Patients with PSC are also susceptible to infections as a result of bacterial colonization of stenosed bile ducts, impaired biliary drainage, and development of liver cirrhosis.^4^ Post-LT immunosuppression may further compound this risk.^5^^,^^6^ It is therefore possible that those with PSC-IBD are at particularly high risk for infections, although few studies have examined this. The aims of this study were to determine the event rates of infections in patients with PSC-IBD compared to those with PSC or IBD alone and identify risk factors that may predict the development of infections.

## Methods

This study was developed according to the Preferred Reporting Items for Systematic Reviews and Meta-Analyses guidelines and Meta-Analysis of Observational Studies in Epidemiology reporting guidelines.^7^^,^^8^ This study was not previously registered but did follow an *a priori* protocol (Table S1).

### Information sources and search strategy

A research librarian conducted a systematic literature search in MEDLINE, Embase, and the Cochrane Central Register of Controlled Trials from inception to September 12, 2024. The search strategy was peer-reviewed by a second information specialist and revised by the authors (Tables S2-S4). Reference lists of included citations and review articles were searched to identify any eligible studies. The grey literature was searched through Scopus, OpenGrey, ClinicalTrials.gov, conference abstracts, and manual web searches.

### Eligibility criteria and study design

We identified primary studies that enrolled adult and pediatric patients with a confirmed diagnosis of PSC and IBD. Studies were included if they reported frequency of infection in patients with PSC-IBD or described risk factors for infection. We included randomized controlled trials and observational studies. Systematic or narrative reviews, meta-analyses, clinical guidelines, case reports, and case series with less than 5 patients were excluded. Non-English studies were excluded if translated English versions were unavailable. Studies reporting the event rate of infections in patients with PSC or IBD were excluded if it was not specified how many of these patients had concurrent PSC-IBD. Three authors (P.T., N.N., and T.M.) independently screened titles and abstracts for eligibility, followed by full-length manuscript review. Disagreements were resolved by discussion and consensus agreement. Two reviewers (P.T., N.N.) independently extracted data regarding study characteristics, patient demographics, PSC and IBD disease characteristics, and infections. Disagreements in data extraction were resolved by consensus agreement.

### Outcomes and definitions

Study outcomes included the rate of site-specific infections or infection-related mortality in patients with PSC and IBD. Site-specific infections included gastrointestinal, hepatobiliary, pulmonary, skin and soft tissue, genitourinary, neurological, and postoperative infections following IBD-related surgery or LT for PSC. Undifferentiated infections such as sepsis, fungemia, and bacteremia were also included. In order to aggregate the total number of infections in patients with PSC-IBD across studies, we calculated the rate of all-cause infection which was defined as the proportion of infectious episodes that occurred in patients with PSC-IBD, regardless of etiology. We also assessed risk factors for developing infection in patients with PSC-IBD.

### Statistical analysis

OpenMeta version 10.10 was used to calculate pooled event rates of all-cause infections and site-specific infections in patients with PSC-IBD. Sensitivity analyses were completed to explore the impact of removing abstracts, case series, and studies published prior to the year 2000 on the results. The *I*^2^ statistic was computed to assess heterogeneity between studies with *I*^2^ above 50% representing substantial heterogeneity.^9^ A Dersimonian-Laird random-effects model was used to generate pooled odds ratios (OR) with 95% confidence intervals (CI) comparing the event rate of infections in patients with PSC-IBD to those with PSC alone and IBD alone.^10^ RevMan Software was used to generate forest plots to summarize the OR comparing event rates between groups on meta-analysis. Two-sided *P*-values were calculated and a *P*-value less than .05 was considered statistically significant. Given lack of available studies, a systematic review without meta-analysis was performed to address our secondary outcome and summarize risk factors for infections in patients with PSC-IBD.

### Quality assessment

Risk of bias of nonrandomized observational studies was independently assessed by 2 authors (T.M., N.N.) using the Newcastle-Ottawa scale (NOS).^11^ Studies were evaluated based on selection of participants, comparability of groups, and assessment of exposures or outcomes. Studies were deemed low risk of bias if the NOS score was greater than 6.^11^ The Cochrane Risk of Bias 2 (RoB 2) tool was used to appraise the quality of randomized studies.^12^ Disagreements in quality assessment were resolved by discussion.

## Results

### Study selection

**Figure 1.**
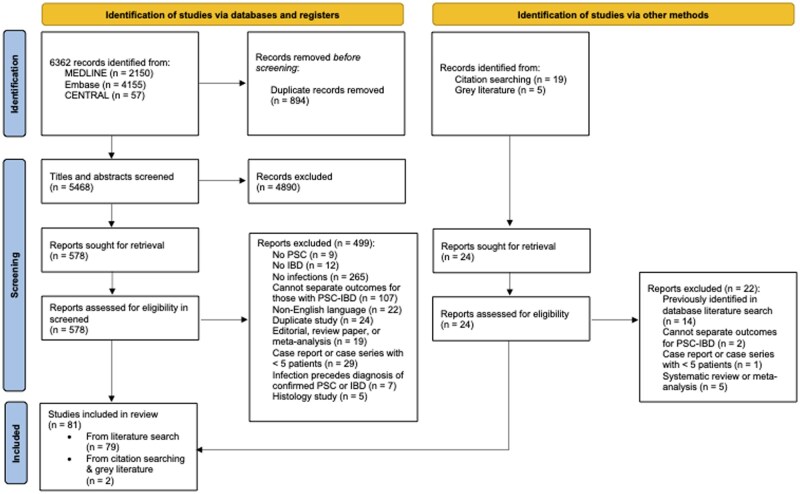
Study flow diagram.

The literature search yielded 6362 records (Figure 1). After excluding 5784 titles and abstracts, 578 studies were assessed in full text. Eighty-one studies were included in the final analysis.^13–93^

### Study characteristics

Sixty-one studies^13–66^^,^^76^^,^^79^^,^^83–87^ were full-text articles and 20^67–75^^,^^77^^,^^78^^,^^80–82^^,^^88–93^ were published as abstracts (Table 1). We included 1 randomized trial^14^ and 80^13^^,^^15–93^ observational studies. Seventy-five studies examined the event rate of all-cause infections in patients with PSC-IBD.^13–81^^,^^83^^,^^86^^,^^87^^,^^91–93^ Six studies investigated risk factors for developing infections in those with PSC-IBD.^82^^,^^84^^,^^85^^,^^88–90^ Six studies assessed both event rate and risk factors for infection in PSC-IBD patients.^13^^,^^15–18^^,^^81^

**Table 1.** Baseline characteristics.

Study Author (year of publication)	Study design	Patient group	Sample size	IBD phenotype			Liver transplant n (%)	Advanced IBD therapy n (%)	IBD-related surgery n (%)	Infectious outcome studied	Follow-up (months)
				CD	UC	IBDU					
**Studies comparing PSC-IBD versus IBD**											
Gorgun (2005)^28^	Case-control	PSC-IBD	65	0	56	9	0 (0)	0 (0)	0 (0)	Fungemia Pelvic sepsis	NR
		IBD	260	0	224	36	0 (0)	0 (0)	0 (0)	Pelvic sepsis	NR
Wasmuth (2010)^32^	Prospective cohort	PSC-UC	11	0	11	0	0 (0)	0 (0)	11 (100)	None	NR
		UC	260	0	260	0	0 (0)	0 (0)	260 (100)	Pelvic sepsis	NR
Ye (2011)^34^	Retrospective cohort	PSC-UC	21	0	21	0	7 (33.3)	0 (0)	0 (0)	Sepsis	80
		UC	63	0	63	0	0 (0)	0 (0)	2 (3.2)	None	85
Pavlides (2014)^30^	Retrospective cohort	PSC-UC-IPAA	21	0	21	0	0 (0)	0 (0)	21 (100)	Pelvic sepsis	NR
		UC-IPAA	79	0	79	0	0 (0)	0 (0)	79 (100)	Pelvic sepsis	NR
Block (2014)^29^	Retrospective case-control	PSC-UC-IPAA	31	0	31	0	11 (35.5)	0 (0)	31 (100)	Pelvic sepsis	NR
		PSC-UC-IRA	17	0	17	0	7 (41.2)	0 (0)	0 (0)	Infection NOS	NR
		UC-IPAA	62	0	62	0	0 (0)	0 (0)	62 (100)	Infection NOS	NR
Bhardwaj^a^ (2016)^67^	Retrospective cohort	PSC-UC	1417	0	1417	0	0 (0)	0 (0)	0 (0)	Sepsis C. Diff	NR
		UC	18 299	0	18 299	0	0 (0)	0 (0)	0 (0)	Sepsis C. Diff	NR
Lascurain (2016)^27^	Retrospective case-control	PSC-IBD	5	32	0	0	6 (16.2)	0 (0)	0 (0)	Cholangitis	NR
		IBD	137	16	123	0	NR	0 (0)	0 (0)	NR	NR
Ramsey^a^ (2016)^82^	Cross sectional	PSC-IBD-CRC	89	NR	NR	NR	0 (0)	0 (0)	0 (0)	NR	NR
		IBD-CRC	3772	NR	NR	NR	0 (0)	0 (0)	0 (0)	NR	NR
Quinn (2022)^31^	Retrospective case-control	PSC-IBD	182	0	175	7	62 (34.1)	0 (0)	0 (0)	C. Diff pouchitis CMV pouchitis	NR
		UC	182	0	177	5	0 (0)	0 (0)	0 (0)	C. Diff pouchitis	NR
Weng (2022)^33^	Retrospective cohort	PSC-UC	12	0	12	0	3 (25)	0 (0)	0 (0)	Sepsis	NR
		UC	38	0	38	0	0 (0)	8 (21.1) Anti-TNF	0 (0)	Pneumonia	NR
**Studies comparing PSC-IBD versus PSC**											
Tobias (1983)^65^	Retrospective cohort	PSC-UC	8	0	8	0	0 (0)	0 (0)	1 (12.5)	Sepsis Cholangitis	NR
Rabinovitz (1990)^24^	Retrospective cohort	PSC-IBD	47	8	39	0	0 (0)	0 (0)	0 (0)	Sepsis	NR
		PSC	19	NR	NR	NR	0 (0)	0 (0)	0 (0)	Sepsis	NR
Martin (1990)^21^	Retrospective cohort	PSC-IBD	88	NR	NR	NR	0 (0)	0 (0)	0 (0)	Cholangitis	NR
		PSC	72	NR	NR	NR	0 (0)	0 (0)	0 (0)	Cholangitis	NR
Miki (1995)^22^	Retrospective cohort	PSC-IBD-LT	31	NR	NR	NR	31 (100)	0 (0)	2 (6.5)	Sepsis	NR
		PSC-LT	24	NR	NR	NR	24 (100)	0 (0)	0 (0)	Sepsis	NR
Vandevrie (2003)	Retrospective cohort	PSC-IBD	18	4	14	0	18 (100)	0 (0)	0 (0)	CMV	62
		PSC	13	NR	NR	NR	13 (100)	0 (0)	0 (0)	CMV	27
Charatcharoenwitthaya (2008)^19^	Prospective cohort	PSC-IBD	22	7	15	0	0 (0)	0 (0)	0 (0)	None	NR
		PSC	20	NR	NR	NR	0 (0)	0 (0)	0 (0)	Cholangitis	NR
Navaneethan (2012)^23^	Retrospective cohort	PSC-UC	167	0	167	0	86 (51.5)	8 (0.6)	92 (55.1)	Cholangitis	NR
		PSC	55	NR	NR	NR	29 (52.7)	NR	1 (1.8)	Cholangitis	NR
Obusez (2013)^17^	Retrospective cohort	PSC-UC-OLT-IPAA	9	0	9	0	9 (100)	0 (0)	0 (0)	Bacteremia Intraabdominal abscess	NR
		PSC-UC-OLT	27	0	27	0	27 (100)	0 (0)	0 (0)	Bacteremia Intraabdominal abscess	NR
		PSC-OLT	30	NR	NR	NR	30 (100)	0 (0)	0 (0)	Bacteremia Intraabdominal abscess	NR
Rupp (2014)^18^	Retrospective cohort	PSC-IBD	108	NR	NR	NR	0 (0)	0 (0)	0 (0)	Biliary candidiasis	NR
		PSC	42	NR	NR	NR	0 (0)	0 (0)	0 (0)	Biliary candidiasis	NR
Venkat (2014)^26^	Retrospective cohort	PSC-IBD	9	NR	NR	NR	9 (100)	0 (0)	1 (11.1)	CMV EBV	NR
		PSC	3	NR	NR	NR	3 (100)	0 (0)	0 (0)	CMV EBV	NR
Lemoinne (2019)^20^	Retrospective cohort	PSC-IBD	27	11	12	4	0 (0)	0 (0)	0 (0)	Cholangitis	NR
		PSC	22	NR	NR	NR	0 (0)	0 (0)	0 (0)	Cholangitis	NR
Irles-Depe (2020)^15^	Retrospective cohort	PSC-IBD-LT	52	7	31	11	52 (100)	6 (11.5) Anti-TNF 1 (1.9) Anti-Integrin	0 (0)	CMV C. Diff	NR
		PSC-LT	35	NR	NR	NR	35 (100)	NR	0 (0)	CMV	NR
Peverelle (2020)^13^	Retrospective cohort	PSC- Mild activity IBD	38	5	30	3	38 (100)	0 (0)	0 (0)	CMV viremia C. Diff CMV colitis Cholangitis	NR
		PSC-moderate activity IBD	34	0	34	0	34 (100)	8 (23.5) Any Biologics	27 (79.4)	CMV viremia C. Diff CMV colitis Cholangitis	NR
		PSC	40	NR	NR	NR	40 (100)	0 (0)	0 (0)	CMV viremia C. Diff CMV colitis Cholangitis	NR
Dahiya^a^ (2024)^93^	Retrospective cohort	PSC-IBD	102	30	72	0	NR	NR	NR	Mortality due to sepsis	NR
		PSC	69	0	0	0	NR	NR	NR	Mortality due to sepsis	NR
**Studies evaluating only patients with PSC-IBD**											
Warren (1966)^66^	Retrospective cohort	PSC-IBD	12	NR	NR	NR	0 (0)	0 (0)	5 (41.7)	Intraabdominal abscess Wound infection	NR
Jorge (1985)^60^	Case series	PSC-IBD	6	2	4	0	0 (0)	0 (0)	0 (0)	Sepsis	NR
Cangemi (1989)^53^	Prospective cohort	PSC-IBD-proctocolectomy	20	0	20	0	0 (0)	0 (0)	20 (100)	Cholangitis	12
		PSC-IBD	25	0	25	0	0 (0)	0 (0)	0 (0)	Cholangitis	NR
Rasmussen (1992)^62^	Retrospective cohort	PSC-UC	11	0	11	0	0 (0)	0 (0)	0 (0)	Sepsis Cholangitis	NR
Sandborn (1993)^14^	Double blind trial	PSC-UC-cyclosporine	16	0	16	0	0 (0)	0 (0)	0 (0)	Cholangitis Candidiasis	NR
		PSC-UC-placebo	10	0	10	0	0 (0)	0 (0)	0 (0)	Candidiasis	NR
Post (1994)^61^	Retrospective cohort	PSC-IBD	24	3	21	0	0 (0)	0 (0)	24 (100)	Sepsis Pelvic sepsis Pneumonia Wound infection	NR
Kartheuser (1996)^50^	Retrospective cohort	PSC-UC proctocolectomy with Brooke ileostomy	32	0	32	0	0 (0)	0 (0)	32 (100)	Pelvic sepsis Cholangitis Wound infection	NR
		PSC-UC-IPAA	40	0	40	0	0 (0)	0 (0)	40 (100)	Pelvic sepsis Wound infection	NR
Khettry (2003)^45^	Retrospective cohort	PSC-IBD-LT	29	4	25	0	29 (100)	0 (0)	0 (0)	Sepsis CMV Hepatitis B Fungemia Cholangitis	NR
Poritz (2003)^54^	Retrospective cohort	PSC-IBD	16	0	16	0	5 (31.3)	0 (0)	16 (100)	CMV Cholangitis	NR
Benavente-Chenhalls (2008)^52^	Retrospective case-control	PSC-UC-laparoscopic IPAA	16	0	16	0	1 (6.3)	1 (6.3) Anti-TNF	16 (100)	Bacteremia Cholangitis Wound infection UTI	NR
		PSC-UC-open IPAA	16	0	16	0	0 (0)	0 (0)	16 (100)	Intraabdominal abscess Wound infection	NR
Mathis (2008)^48^	Retrospective cohort	PSC-IBD-LT	32	0	32	0	32 (100)	0 (0)	32 (100)	Wound infection	NR
Steinhagen^a^ (2009)^80^	Prospective cohort	PSC-UC	16	0	16	0	6 (37.5)	0 (0)	16 (100)	Intraabdominal abscess	NR
Mathis (2011)^46^	Retrospective cohort	PSC-IBD	100	0	100	0	14 (14)	2 (2) Anti-TNF	100 (100)	Bacteremia Wound infection UTI Cholangitis	1
Singal (2011)^64^	Case series	PSC-IBD	14	0	13	1	0 (0)	0 (0)	0 (0)	Sepsis	42.7
Hanouneh (2012)^44^	Case-control	PSC-IBD-LT	43	21	22	0	43 (100)	0 (0)	12 (27.9)	CMV	NR
		PSC-IBD	30	NR	NR	NR	0 (0)	0 (0)	18 (60)	NR	NR
		LT	43	NR	NR	NR	43 (100)	0 (0)	0 (0)	CMV	NR
Indriolo^a^ (2012)^77^	Retrospective cohort	PSC-IBD	29	NR	NR	NR	13 (44.8)	5 (17.2) Anti-TNF	0 (0)	Cholangitis Genital herpes Molluscum contagiosum	NR
Lian (2012)^49^	Retrospective cohort	PSC-IBD	23	1	22	0	0 (0)	0 (0)	23 (100)	Pelvic sepsis Wound infection	NR
Mohabbat (2012)^42^	Retrospective cohort	PSC-IBD	8	5	3	0	8 (100)	8 (100) Anti-TNF	6 (75)	C. Diff Cryptosporidiosis Pneumonia Candidiasis	NR
Drastich (2013) ^69^	Retrospective cohort	PSC-UC	72	0	72	0	72 (100)	0 (0)	3 (4.2)	CMV colitis	80
Indriolo^a^ (2013)^71^	Retrospective cohort	PSC-IBD-LT	4	NR	NR	NR	4 (100)	0 (0)	0 (0)	Molluscum contagiosum	NR
Treeprasertsuk (2013)^51^	Retrospective cohort	PSC-IBD	104	0	104	0	13 (12.5)	0 (0)	104 (100)	Intraabdominal abscess Cholangitis Wound infection	NR
Barnabas^a^ (2014)^68^	Retrospective cohort	PSC-UC	7	0	7	0	7 (100)	0 (0)	0 (0)	Esophageal candidiasis Campylobacter/norovirus Cholangitis	NR
Kochhar^a^ (2015)^72^	Case-control	PSC-IBD	10	NR	NR	NR	10 (20)	10 (20) Anti-TNF	0 (0)	Postoperative infection	NR
Schnitzler (2015)^63^	Prospective cohort	PSC-IBD	24	3	21	0	24 (100)	3 (12.5) Anti-TNF	0 (0)	Sepsis	93
Bramuzzo (2016)^35^	Retrospective cohort	PSC-IBD	28	4	23	1	0 (0)	0 (0)	0 (0)	Cholangitis	NR
		PSC-AIH-IBD	15	1	13	1	0 (0)	0 (0)	0 (0)	Cholangitis	NR
Fain^a^ (2017)^70^	Retrospective cohort	PSC-IBD	19	4	15	0	19 (100)	1 (5.3) Anti-TNF	0 (0)	C. Diff colitis CMV colitis	NR
Altwegg (2018)^40^	Retrospective case series	UC-IRA	51	0	51	0	0 (0)	0 (0)	51 (100)	NR	NR
Lawlor^a^ (2018)^92^	Retrospective cohort	PSC-IBD-biologic exposed	29	10	18	1	NR	20 (69) Anti-TNF 9 (31) Anti-Integrin	NR	Infections Cholangitis C diff	NR
		PSC-IBD-biologic naive	9	2	7	0	NR	0 (0)	NR		
Mouchli (2018) ^47^	Retrospective cohort	PSC-IBD-LT	151	NR	NR	NR	151 (100)	0 (0)	0 (0)	CMV	NR
		PSC-*de novo* IBD-LT	22	NR	NR	NR	22 (100)	0 (0)	0 (0)	CMV	NR
Nedelkopoulou (2018)^38^	Retrospective cohort	PSC-AIH-IBD	9	1	8	0	1 (11.1)	9 (100) Anti-TNF	0 (0)	CMV Cholangitis	NR
Parekh (2018)^43^	Case series	PSC-IBD	5	2	3	0	5 (100)	5 (100) Anti-TNF	1 (20)	Bacteremia CMV Esophageal candidiasis C. Diff Cholangitis Hepatic abscess Pneumonia Cellulitis	NR
Peverelle^a^ (2018)—ECCO^75^	Retrospective case series	PSC-IBD	5	1	4	0	0 (0)	5 (100) Anti-integrin	0 (0)	Colitis	6
Peverelle^a^ (2018)—JGH^74^	Retrospective cohort	PSC-IBD	70	4	63	3	70 (100)	0 (0)	27 (38.6)	N/A	5
Al Draiweesh (2019)^41^	Case series	PSC-IBD	16	10	6	0	16 (100)	5 (31.6) Anti-TNF 9 (56.3) Anti-integrin 1 (5.56) Anti-IL12/23	0 (0)	CMV colitis John Cunningham virus C. Diff Perianal abscess Cholangitis Pneumonia	NR
Caron (2019)^56^	Retrospective cohort	PSC-IBD	75	26	49	0	3 (4)	0 (0)	0 (0)	NR	19.2
Christensen (2019)^55^	Retrospective cohort	PSC-IBD	34	16	18	0	9 (26.4)	27 (79.4) Anti-integrin	0 (0)	Cholangitis URTI	NR
Guerra (2019)^59^	Retrospective cohort	PSC-IBD	277	79	197	0	35 (12.6)	0 (0)	0 (0)	Cellulitis Respiratory infection	67
Mousa^a^ (2019)^73^	Retrospective cohort	PSC-IBD	30	10	20	0	30 (100)	17 (56.7) Anti-TNF 20 (66.7) Anti-integrin 4 (13.3) Anti-IL12/23	0 (0)	C. Diff colitis cholangitis	24.6
Rosenblatt^a^ (2019)^81^	Retrospective cohort	PSC-IBD	126	33	93	0	0 (0)	29 (23) Any biologics	30 (23.8)	Cholangitis	NR
Tse (2019)^78^	Retrospective cohort	PSC-IBD	48	16	29	2	10 (20.8)	0 (0)	14 (29.2)	C. Diff colitis Cholangitis	NR
Hedin (2020)^58^	Retrospective cohort	PSC-IBD	186	NR	NR	NR	0 (0)	0 (0)	0 (0)	Cholangitis	NR
Laborda (2020)^37^	Retrospective cohort	PSC-IBD	37	11	22	4	1 (2.7)	37 (100) Anti-TNF	0 (0)	Cholangitis	NR
Lynch (2020)^57^	Retrospective cohort	PSC-IBD	102	30	66	6	0 (0)	66 (64.7) Anti-TNF 102 (100) Anti-integrin	0 (0)	Cholangitis	NR
Hensel (2021)^36^	Retrospective cohort	PSC-IBD	82	12	45	25	0 (0)	17 (20.7) Any biologics	0 (0)	Cholangitis	NR
Kulkarni (2021)^16^	Retrospective cohort	PSC-IBD	162	29	124	9	0 (0)	28 (17.3) Anti-TNF 14 (8.6) Anti-integrin	0 (0)	Cholangitis	NR
Osiecki (2021)^39^	Retrospective cohort	PSC-UC-OLT	17	0	17	0	17 (100)	0 (0)	0 (0)	CMV EBV	NR
Baston Rey^a^ (2022)^88^	Retrospective cohort	IBD patients with solid organ transplant (including those with PSC)	106	47	58	1	58 (54.7)	19 (17.9) Anti-TNF 10 (9.4) Anti-integrin 2 (1.9) IL12/23 inhibitor	NR	PSC as risk factor for severe infection following solid organ transplant	NR
Medina-Morales^a^ (2022) ^9^	Retrospective cohort	PSC	153	NR	NR	NR	NR	NR	NR	Risk factors for development of acute cholangitis in PSC	NR
Catassi (2023)^85^	Retrospective case-control	Pediatric PSC-IBD	69	4	60	5	4 (5.8)	0 (0)	1 (1.4)	Time to infective cholangitis	NR
Dunleavy (2023)^87^	Retrospective cohort	PSC-IBD-IPAA	99	6	93	0	7 (7.1)	16 (16.2) Biologics 0 (0) Small molecule	99 (100) Total procto-colectomy with IPAA	Abscess C Diff pouchitis	NR
		PSC-IBD-subtotal colectomy	26	13	13	0	3 (11.5)	8 (30.8) Biologics 0 (0) Small molecule	26 (100) Subtotal colectomy with ileosigmoid or ileorectal anastomosis		
Lee (2023)^84^	Retrospective cohort	PSC-IBD post-liver transplant	1801	372	1360	69	1801 (100)	NR	NR	Sepsis Sepsis-related mortality All cause infection-related mortality	NR
Maspero (2023)^86^	Retrospective cohort	PSC-IBD-IPAA	137	4	133	0	0 (0)	NR	137 (100) TAC with IPAA	Infection Pelvic abscess UTI	NR
		PSC-IBD-IPAA-LT	23	0	23	0	23 (100)	NR	23 (100) TAC with IPAA		
Sayed (2023)^79^	Retrospective cohort	PSC-IBD	72	11	61	0	5 (6.9)	10 (13.9) Anti-TNF 5 (6.9) Anti-integrin 6 (8.3) Anti-IL12/23 1 (1.4) Small molecule	16 (22.2)	Cholangitis	51
Schregel (2023)^76^	Retrospective cohort	Nontransplant IBD patients with large-duct PSC who had received ≥4 weeks tofacitinib	42	NR	NR	NR	0 (0)	42 (100) Tofacitinib	NR	Bacterial cholangitis Salmonella associated colitis	NR
Holvoet^a^ (2024)^91^	Retrospective case series	PSC-IBD-LT	58	12	44	2	58 (100)	24 (38) UST 40 (62) VDZ	12 (18.8) IPAA	CMV infection	NR
Karime (2024)^83^	Retrospective cohort	IBD-PSC cirrhosis with TIPS placement	7	0	7	0	0 (0)	NR	7 (100) Colorectal surgery after TIPS placement	Systemic infection	NR
Malhi^a^ (2024)^90^	Retrospective cohort	PSC-IBD-LT	99	NR	NR	NR	99 (100)	NR	NR	Risk factors for infection in PSC-LT patients	NR

Abbreviations: AIH, autoimmune hepatitis; CD, Crohn’s disease; C. Diff, clostridioides difficile; CMV, cytomegalovirus; CRC, Colorectal Cancer; EBV, Epstein-Barr virus; IBD, inflammatory bowel disease; IBD-U, IBD unclassified; IPAA, ileal pouch-anal anastomosis; IRA, ileorectal anastomosis; LT, liver transplant; NOS, not otherwise specified; NR, not reported; OLT, orthotopic liver transplantation; PSC, primary sclerosing cholangitis; TAC, total abdominal colectomy; TIPS, transjugular intrahepatic portosystemic shunt; UC, ulcerative colitis; URTI, upper respiratory tract infection; UST, ustekinumab; UTI, urinary tract infection; VDZ, vedolizumab.

a Denotes that study is abstract.

#### PSC-IBD

The pooled event rate of all-cause infection was 25.1% (95% CI, 17.0%-33.2%, *I*^2^ = 99.2%) in those with PSC-IBD (5481 patients, 75 studies).^13–81^^,^^83^^,^^84^^,^^87^^,^^91–93^ Studies reported a follow-up ranging anywhere from 1 month^49^^,^^83^ to 13.7 years.^87^ The pooled event rate of all-cause infections in pediatric patients with PSC-IBD (234 patients, 7 studies) was 24.6% (95% CI, 11.6%-37.6%, *I*^2^ = 93.7%)^26^^,^^27^^,^^35–39^ compared to 24.9% (95% CI, 16.1-33.7, *I*^2^ = 99.2%) in adult patients.^13–25^^,^^28–34^^,^^40–81^^,^^83^^,^^84^^,^^87^^,^^91–93^ Sensitivity analyses did not impact these pooled event rates (Table S5).

#### PSC-IBD versus IBD

**Figure 2.**
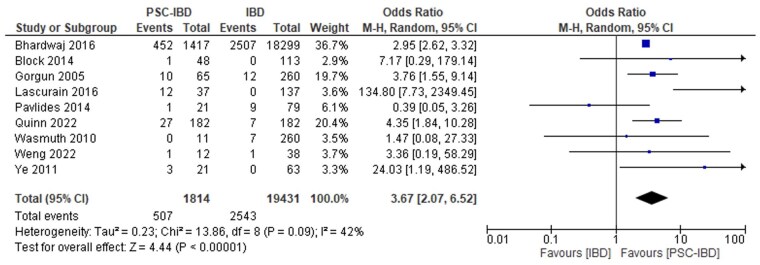
Event rate of all-cause infections in patients with primary sclerosing cholangitis and inflammatory bowel disease (PSC-IBD) compared to patients with IBD.

**Figure 3.**
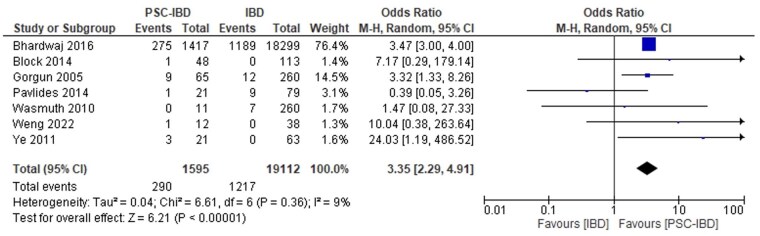
Event rate of sepsis in patients with primary sclerosing cholangitis and inflammatory bowel disease (PSC-IBD) compared to those with IBD.

**Figure 4.**
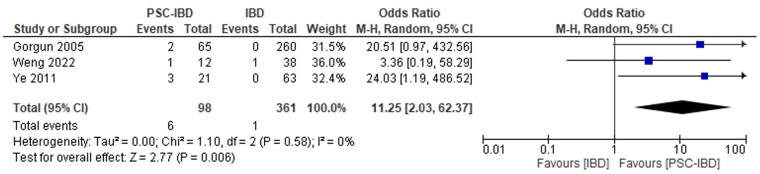
Event rate of infection-related mortality in patients with primary sclerosing cholangitis and inflammatory bowel disease (PSC-IBD) compared to those with IBD.

Patients with PSC-IBD had higher odds of all-cause infection (which included fungemia, pelvic sepsis, clostridioides difficile [C. difficile], cytomegalovirus [CMV], community-acquired pneumonia, and cholangitis) compared to those with IBD (OR 3.67, 95% CI, 2.07-6.52, *I*^2^ = 41.9%, Figure 2).^27–34^^,^^67^ Patients with PSC-IBD also had increased odds of sepsis (OR 3.35, 95% CI, 2.29-4.91, *I*^2^ = 9.1%, Figure 3)^28–30^^,^^32–34^^,^^67^ and infection-related mortality (OR 11.25, 95% CI, 2.03-62.37, *I*^2^ = 0, Figure 4)^28^^,^^33^^,^^34^ compared to those with IBD.

##### Postoperative infections following colorectal surgery in PSC-IBD

The pooled event rate of postoperative infections in 869 PSC-IBD patients undergoing colorectal surgery for IBD-related complications was 19.6% (95% CI, 12.1%-27.1%, *I*^2^ = 92.5%).^17^^,^^28–30^^,^^32^^,^^46^^,^^48–54^^,^^80^^,^^86^^,^^87^ There was no significant difference in postoperative infections following IBD-related surgery in patients with PSC-IBD compared to those with IBD alone (OR 2.15, 95% CI, 0.64-7.21, *I*^2^ = 33%, Figure S4).^28–30^^,^^32^ The most common surgery studied was restorative proctocolectomy with ileal pouch-anal anastomosis (IPAA). Pelvic sepsis^30^^,^^32^ was the most common postoperative infection followed by cholangitis.^27^

One study investigated risk factors for postoperative infections in IBD patients undergoing surgery for colorectal cancer.^82^ Concurrent PSC was associated with increased development of postoperative infections both on univariable (OR 4.03, 95% CI, 1.31-12.4) and multivariable analysis (OR 4.57, 95% CI, 1.46-14.31).^82^ Meanwhile Rosenblatt et al^81^ found that prior IBD surgery did not predict development of the first episode of acute bacterial cholangitis in patients with PSC-IBD (Hazard ratio (HR) 0.58, 95% CI, 0.15-2.15).

##### Infections associated with advanced IBD therapies in PSC-IBD

The pooled event rate of all-cause infection in PSC-IBD patients receiving advanced therapies (biologics or small molecules) was 16.2% (95% CI, 11.0%-21.4%, *I*^2^ = 76.5%).^37^^,^^38^^,^^40^^,^^55–58^^,^^68^^,^^72^^,^^76^^,^^78^^,^^79^^,^^91^^,^^93^ The most commonly administered biologic was vedolizumab (*n* = 339) with a pooled all-cause infection event rate of 15.8% (95% CI, 7.4%-24.1%, *I*^2^ = 83.4%),^37^^,^^55–57^^,^^78^^,^^91^ followed by anti-TNFα therapies which were associated with an all-cause infection event rate of 7.7% (95% CI, 2.5%-12.9%, *I*^2^ = 3.3%).^38^^,^^42^^,^^58^^,^^68^ Of the 682 patients with PSC-IBD who were receiving advanced IBD therapies, 99 received prior LT and were on additional immunosuppression.^42^^,^^68^^,^^72^^,^^78^^,^^91^ The pooled event rate of all-cause infections in this post-LT population was increased at 37.0% (95% CI, 27.6%-46.3%, *I*^2^ = 0%).^42^^,^^68^^,^^72^^,^^78^^,^^91^

Two studies examined the impact of IBD therapies as potential risk factors for infection in PSC-IBD patients.^16^^,^^81^ In 1 study, neither the use of biologics (HR 1.56, 95% CI, 0.56-4.34) nor immunomodulators (HR 1.23, 95% CI, 0.34-4.43) predicted the development of acute bacterial cholangitis in patients with PSC-IBD.^81^ Meanwhile, Kulkarni et al^16^ found that PSC-IBD patients receiving anti-TNFα therapy had a more than 3-fold odds of developing bacterial cholangitis (OR 3.45, 95% CI, 1.54-7.76), with 50% of patients developing cholangitis within 36 months of starting therapy. In the same study, vedolizumab was not associated with increased bacterial cholangitis (OR 0.71; 95% CI, 0.19-2.72), and immunomodulators conferred a protective effect (OR 0.23, 95% CI 0.05-0.77).^16^

#### PSC-IBD versus PSC

**Figure 5.**
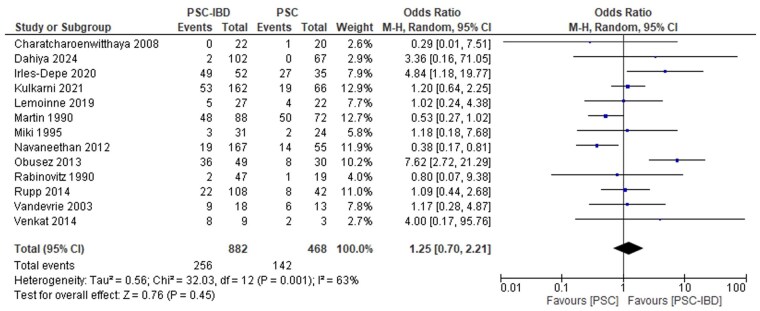
Event rate of all-cause infections in patients with primary sclerosing cholangitis and inflammatory bowel disease (PSC-IBD) compared to patients with PSC.

There was no difference in the odds of all-cause infection (OR 1.25, 95% CI, 0.71-2.21, *I*^2^ = 62.4%, Figure 5)^15–26^^,^^93^ or infection-related mortality (OR 0.85, 95% CI, 0.27-2.67, *I*^2^ = 0%, Figure S1)^17^^,^^24^^,^^25^^,^^93^ in patients with PSC-IBD compared to those with PSC alone. There was no difference in the frequency of cholangitis between patients with PSC-IBD and those with PSC (OR 0.66, 95% CI, 0.39-1.13, *I*^2^ = 39.5%, Figure S5).^16^^,^^19–21^^,^^23^ Similar results were reported in 3 observational studies, where the presence of IBD was not associated with cholangitis^16^^,^^81^ or candidiasis on bile fungal cultures obtained from PSC-IBD patients (HR 0.7, 95% CI 0.3-2.1).^18^

##### Post-LT infections

The pooled event rate of all-cause infections in PSC-IBD patients following LT was 35.8% (95% CI, 23.0-48.6%, *I*^2^ = 97.3%).^15^^,^^17^^,^^22^^,^^26^^,^^39–47^^,^^68–75^^,^^86^^,^^91^ There was no significant difference in all-cause infections across PSC-IBD patients undergoing LT compared to those who did not receive LT (OR 2.92, 95% CI, 0.36-23.69, *I*^2^ = 67.3%, Figure S2).^44^^,^^78^^,^^86^ Compared to those with PSC undergoing LT, PSC-IBD patients undergoing LT had an over 3-fold increased odds of developing infection (OR 3.31, 95% CI, 1.86-5.88, *I*^2^ = 4.9%, Figure S3).^13^^,^^15^^,^^17^^,^^22^^,^^26^^,^^47^ The most common post-LT infection was CMV with a pooled event rate of 23.5% (95% CI, 15.1%-31.9%, *I*^2^ = 79.2%),^13^^,^^15^^,^^26^^,^^39^^,^^41^^,^^43–45^^,^^47^^,^^91^ followed by Epstein-Barr virus,^26^^,^^39^ C. difficile colitis,^13^^,^^40^^,^^41^^,^^69^^,^^70^ sepsis,^22^^,^^45^ intra-abdominal abscess,^17^^,^^26^ and bacteremia.^17^

The pooled event rate of CMV infection across 554 patients with PSC-IBD was 20.8% (95% CI, 12.8%-28.8%, *I*^2^ = 84.8%), with 87.9% having prior LT.^13^^,^^15^^,^^25^^,^^26^^,^^38^^,^^39^^,^^41^^,^^43–45^^,^^47^^,^^54^^,^^63^^,^^91^ There was an over 2-fold increased odds of CMV infection in those with PSC-IBD compared to those with PSC alone (OR 2.40, 95% CI 1.04-5.51, *I*^2^ = 0%, Figure S6).^15^^,^^25^^,^^26^ Two^15^^,^^26^ of these studies examined risk factors for CMV post-LT and identified that the presence of IBD and donor CMV status were associated with reactivation of CMV infection in PSC-IBD patients.^15^

Three studies examined risk factors for infection following solid organ transplant in patients with PSC and IBD.^84^^,^^88^^,^^90^ One study noted that PSC was a risk factor for severe infection following transplant in patients with pre-existing IBD (OR 3.3, 95% CI, 1.3-8.6).^88^ Meanwhile, in a study of patients with PSC undergoing LT, presence of IBD was not associated with development of infections at 30 days (OR 1.01, 95% CI, 0.98-1.04).^90^ One study examined the role of IBD subtype on infection-related mortality following LT in patients with PSC and noted higher rates of infection-related death in those with PSC-CD compared to those with PSC alone or PSC-UC.^84^

Two studies reported mixed results regarding the association of post-LT IBD disease activity and infectious complications in patients with PSC-IBD.^13^^,^^47^ One study found that moderate or severe IBD was associated with an increased risk of cholangitis and C. difficile colitis following LT on univariable analysis but not multivariable analysis.^13^ Meanwhile, another study identified similar rates of post-LT CMV infection in PSC-IBD patients regardless of whether their IBD course was stable, progressed, or improved after LT.^47^

### Risk of bias assessment

Twenty-five (37.1%) cohort studies^13^^,^^15^^,^^17^^,^^19^^,^^20^^,^^23^^,^^30–32^^,^^35^^,^^42^^,^^47^^,^^48^^,^^50^^,^^51^^,^^57^^,^^59^^,^^71^^,^^78^^,^^79^^,^^81^^,^^84^^,^^85^^,^^89^^,^^90^ and 5 (85.7%) case-control studies^27^^,^^28^^,^^44^^,^^52^^,^^72^ demonstrated low risk of bias on the NOS (Tables S6 and S7). A single randomized controlled trial^14^ also revealed low risk of bias on the Cochrane RoB 2 tool (Table S8). A sensitivity analysis was completed where only those studies at low risk of bias were included, with no difference in the overall pooled infection rate (Table S5).

## Discussion

We demonstrate that patients with PSC-IBD have higher rates of all-cause infection, sepsis, and mortality compared to those with IBD, but not those with PSC alone. Our review suggests that in those with concomitant PSC-IBD, the diagnosis of PSC may modulate the risk of adverse infection-related outcomes.

Primary sclerosing cholangitis is a cholestatic liver disease characterized by progressive biliary stricturing that is often complicated by superimposed infection.^1^^,^^2^ With no disease-modifying therapies available to halt progression of fibrosis, patients may develop ascending cholangitis from biliary obstruction.^1–3^ While the pathogenesis of PSC may explain the increased occurrence of cholangitis in PSC-IBD patients, this mechanism does not fully account for the higher rates of noncholangitic infections in this group. This implies that there are likely other features of PSC modulating the infection risk observed. For example, cirrhosis is a complication of PSC that confers an almost 5-fold higher risk of infection than that of the general population due to immune dysfunction.^94^ Autoimmune hepatitis (AIH) is also reported in 6%-14% of adults with PSC and typically requires prolonged corticosteroid treatment which has a dose-dependent association with opportunistic infections.^95^^,^^96^ Only 2 studies in this review explored infectious complications in patients with PSC-IBD and concomitant cirrhosis or AIH, with sample sizes ranging from 7 to 15, while the remaining studies did not consistently report on the presence or absence of cirrhosis or AIH. Finally, there is emerging evidence that patients with PSC and PSC-IBD may have a unique gut microbiome compared to healthy controls and those with IBD alone.^97^ Gut dysbiosis has been linked to immune dysregulation, making it plausible that the gut-liver axis in patients with PSC-IBD may be implicated in their susceptibility to infection.^98^

Patients with PSC-IBD undergoing LT appear to represent a distinct population with increased risk of infections, especially CMV, compared to those with PSC alone. Although LT is a treatment option for patients with PSC who experience recurrent cholangitis and/or cirrhosis (both established risk factors for infection), LT seems to be an independent risk factor for infection in this patient population. For example, it has been shown that those with LT may develop infection rates as high as 45% within the first 6 months postoperatively.^5^^,^^6^ The high rates of posttransplant infection are multifactorial and related to the use of postoperative lines/catheters, intensive care admissions, surgical techniques, and donor characteristics.^5^ Furthermore, individuals who undergo orthotopic liver transplantation are treated with long-term immunosuppressive medication such as calcineurin inhibitors, antimetabolites, and/or steroids which are associated with numerous infectious complications. When combined with advanced therapies for IBD, we hypothesize an increased risk of opportunistic and invasive infections in patients with PSC-IBD, which would explain the predominance of CMV infection in this patient population. We hypothesize that the additive effects of malnutrition and gut dysbiosis seen in IBD patients may further potentiate the post-LT infection risk in PSC-IBD patients, potentially explaining the higher risk of infection in this group compared to those receiving LT for PSC alone.

Although PSC-IBD patients had a higher occurrence of all-cause infection compared to those with IBD alone, the risk of postoperative infections following colorectal surgery was overall low and similar between groups. Pelvic sepsis after IPAA was the most studied infection, which generally has a low incidence with modern-day surgical techniques.^99^ Furthermore, patients selected to undergo IPAA generally have well-controlled disease at the time of surgery without any significant comorbidities such as advanced liver disease. Therefore, these studies may represent a healthier subset of IBD and PSC-IBD patients with similar baseline risk.

The association between biologic use and infection risk in patients with PSC-IBD remains unclear. In this systematic review, we did not identify any head-to-head studies directly comparing the event rate of infection in patients with PSC-IBD to those with isolated IBD based on immunosuppressive regimen. Although infection is an established risk of immunomodulators and anti-TNFα drugs in the general population, the included studies reported mixed results in those with PSC-IBD.^16^^,^^82^ As the therapeutic armamentarium for IBD continues to expand, future studies should assess the risk of infection with advanced therapies in those with PSC-IBD.

Despite comprehensively synthesizing the data regarding infections in patients with PSC-IBD, there are several limitations. Many studies had a retrospective design with incomplete reporting of data such as immunosuppressive medications, comorbidities, duration of follow-up, and disease control, which limits our ability to ascertain the impact of these variables on short- and long-term infection risk in patients with PSC-IBD. Studies that did report on these variables had significant heterogeneity, including the selection of patients with less aggressive forms of PSC (ie, those with small duct PSC or those without advanced fibrosis) which could skew the overall results. However, we tried to account for this limitation with the use of subgroup analyses. The follow-up period also varied from 1^49^ to 80^34^ months, meaning long-term infections may have potentially been missed. It is possible that the rates of infections may be under reported as infection was rarely the primary outcome in the included studies but rather reported as an ancillary finding with no *a priori* definition. This is especially true in patients with PSC who are frequently treated with prophylactic antibiotics, despite lacking evidence, which may mask potential infections.

## Conclusion

Patients with PSC-IBD appear to be at a higher risk of infection and infection-related mortality compared to those with IBD alone, but not PSC alone. Our review suggests that the diagnosis of PSC may be a modifying risk factor for infection in patients with IBD, although further research is needed to understand the disease characteristics associated with PSC leading to this heightened susceptibility. Healthcare providers should maintain a low threshold to screen PSC-IBD patients for infections and early recognition of high-risk symptoms should prompt immediate treatment in order to reduce complications of sepsis and mortality.

## Supplementary Material

gwaf023_Supplemetary_Data

## Data Availability

The data underlying this article are available in the article and in its online supplementary material.
