# Supplementary material for: Infections in primary sclerosing cholangitis and inflammatory bowel disease: a systematic review and meta-analysis
Source: J Can Assoc Gastroenterol. 2025 Sep 3;8(5):163–78. doi: 10.1093/jcag/gwaf023 (PMC12551746; doi:10.1093/jcag/gwaf023)
Supplement: gwaf023_Supplemetary_Data [file gwaf023_supplemetary_data.zip › Supplementary Tables (no markup).docx]

**Supplementary Tables**

**Supplementary Table 1: Protocol for “Infections in Primary Sclerosing Cholangitis and Inflammatory Bowel Disease: A Systematic Review & Meta-Analysis”**

| **Title** | **Infections in Primary Sclerosing Cholangitis and Inflammatory Bowel Disease: A Systematic Review & Meta-Analysis** |
| --- | --- |
| **Background** | Primary sclerosing cholangitis (PSC) is a chronic inflammatory condition of the biliary tract that results in fibrosis of the intra- and/or extrahepatic bile ducts. Patients with PSC often have other immune-mediated comorbidities such as inflammatory bowel disease (IBD), which consists of both ulcerative colitis (UC) and Crohn’s Disease (CD). Up to 80% of PSC patients may develop UC while the overlap of PSC and CD occurs less frequently at a rate of 2-14%.  Those with concurrent PSC and IBD (PSC-IBD) represent a unique cohort of patients with an elevated risk of complications such as colorectal cancer, dysplasia, and post-operative pouchitis following colectomy. While these risks have been well documented in the literature, the risk of infectious complications in patients with PSC-IBD remains unclear.  Infection is an established risk in patients with IBD, both due to the underlying pathophysiology of the disease and the immunosuppressive therapy used to manage it. Patients with PSC are also vulnerable to infection, specifically cholangitis, due to cholestasis and bacterial colonization of the stenosed bile ducts. It would thus follow that those with PSC-IBD would be a particularly high-risk group for infectious complications, although few studies have actually examined the prevalence of infection in these patients. Furthermore, no systematic review or meta-analysis has been conducted to date examining the risk factors that may predict development of infectious complications. As such, this systematic review seeks to bridge an important gap in the literature by identifying the types of infections that may occur in patients with PSC-IBD and risk factors that may predict infections in this high-risk population. |
| **Research Objectives** | This review will address the following two research questions:  1. What is the incidence of all-cause and site-specific infections in patients with PSC-IBD and the odds of these infections compared to those with PSC alone and IBD alone?  2. What are the risk factors for developing infectious complications in patients with PSC-IBD? |
| **Search Strategy** | We will search the following electronic databases from inception: PubMed, Medline, Embase, and Cochrane Central Register of Controlled Trials. Reference lists of full-length manuscripts and prior systematic reviews will also be searched for studies that meet our inclusion criteria. Although systematic reviews will be excluded from the final analysis, relevant reviews will be hand-searched for relevant studies. Finally, we will search abstracts from gastroenterology conferences including Digestive Diseases Week, American College of Gastroenterology, and the European Crohn’s and Colitis Organization for applicable studies from 2010 to 2024.  Two authors will independently review the titles/abstracts, followed by the full-length manuscripts for inclusion. A third reviewer will be consulted if any disagreements arise regarding study eligibility. Studies that meet inclusion criteria after both title/abstract and full-text screening will be included in the final qualitative and quantitative analysis. A Kappa value will be calculated to assess agreement between reviewers.  **Years to Search:**  Inception to Present |
| **Inclusion & Exclusion Criteria** | We will include all studies with adult or pediatric patients with primary sclerosing cholangitis (PSC) and inflammatory bowel disease (IBD) – either Crohn’s disease (CD) or ulcerative colitis (UC) – that examine:  1) Incidence of Infections   - Bacterial or viral or fungal - Sepsis or septic shock - Cholangitis or biliary sepsis - Respiratory infections - Gastrointestinal infections - Genitourinary infections - Skin and soft tissue infections - Central nervous system infections   **OR**  2) Risk factors that may influence development of infections including, but not limited to:   - Medication use such as biologics, corticosteroids, and other immunosuppressive therapy - Age - Gender - Comorbidities such as frailty - Liver transplant - Surgery (such as colectomy and ileal pouch-anal anastomosis)   **Inclusion Criteria:**  This review will include all observational cohort studies, controlled trials, case-control studies, case reports, and case series that include pediatric or adult patients with an established diagnosis of PSC and IBD. Eligible studies must report on an infectious complication or risk factor for its development. Both full-length articles and abstracts will be included.  **Exclusion Criteria:**   - Patient does not have confirmed diagnosis of PSC - Patient does not have confirmed diagnosis of IBD - No report of infections - Review articles - Editorials - Systematic reviews / meta-analyses - Non-English language |
| **Outcomes** | **Primary Outcome**  The primary outcome of this study is to determine whether the risk of infections is higher in patients with inflammatory bowel diseases and PSC, compared to those with PSC alone or IBD alone  **Secondary Outcomes**   - To assess the rates of all-cause and site-specific infections in patients with PSC and IBD - To assess risk factors for all-cause and site-specific infections in patients with PSC and IBD   - Specific risk factors to be assessed include:     - Biologic use and immunomodulator use     - Disease severity     - Comorbidities     - Transplant status |

**Supplementary Table 2: MEDLINE Search Strategy**

| **#** | **Query** | **Results from 14 Sep 2024** |
| --- | --- | --- |
| 1 | Cholangitis, Sclerosing/ | 4882 |
| 2 | (primary adj3 sclerosing adj3 cholangitis).ab,hw,kf,ti. | 6244 |
| 3 | exp Inflammatory Bowel Diseases/ | 102662 |
| 4 | (inflammatory adj3 bowel adj3 diseas*).ab,hw,kf,ti. | 76097 |
| 5 | Colitis/ | 25125 |
| 6 | pancolitis.ab,hw,kf,ti. | 920 |
| 7 | (indeterminate adj2 colitis).ab,hw,kf,ti. | 856 |
| 8 | (crohn disease or crohns disease or crohn's disease).ab,hw,kf,ti. | 68692 |
| 9 | exp proctitis/ or exp proctocolitis/ | 3322 |
| 10 | 1 or 2 | 7704 |
| 11 | 3 or 4 or 5 or 6 or 7 or 8 or 9 | 155547 |
| 12 | 10 and 11 | **2150** |

**Supplementary Table 3: Embase Search Strategy**

| **#** | **Query** | **Results from Sep 14, 2024** |
| --- | --- | --- |
| 1 | primary sclerosing cholangitis/ | 12467 |
| 2 | colitis/ or crohn disease/ or pancolitis/ or proctitis/ or proctocolitis/ or ulcerative colitis/ | 237602 |
| 3 | (indeterminate adj2 colitis).mp. [mp=title, abstract, heading word, drug trade name, original title, device manufacturer, drug manufacturer, device trade name, keyword heading word, floating subheading word, candidate term word] | 2308 |
| 4 | inflammatory bowel disease/ | 68176 |
| 5 | 2 or 3 or 4 | 269628 |
| 6 | 1 and 5 | 4155 |

**Supplementary Table 4: EBM Reviews Cochrane Central Register of Controlled Trials (CENTRAL) Search Strategy**

| **#** | **Query** | **Results from 14 Sep 2024** |
| --- | --- | --- |
| 1 | "primary sclerosing cholangitis" | 265 |
| 2 | "inflammatory bowel disease" OR "inflammatory bowel diseases" OR "crohn's" OR "Crohn" OR "crohns" OR "ulcerative colitis" OR "colitis" OR "proctitis" OR "proctosigmoiditis" OR "IBD" | 102 |
| 3 | 1 AND 2 | 57 |

**Supplementary Table 5**: **Sensitivity analyses assessing impact of various subgroups and removing abstracts/case series on incidence of all-cause infections in patients with PSC-IBD**

| **Group Studied** | **Number of Studies Included (n)** | **Patients Included (n)** | **Pooled Event Rate of All-Cause Infections (%)** | **95% Confidence Interval (CI)** | **I^2^** |
| --- | --- | --- | --- | --- | --- |
| All adult and pediatric patients with PSC-IBD^13-81,83,86,87,91-93^ | 75 | 5481 | 25.1 | 17.0-33.2 | 99.2 |
| Adult patients with PSC-IBD^13-25,28-34,40-81, 83,84,87,91-93^ | 68 | 5235 | 24.9 | 16.1-33.7 | 99.2 |
| Pediatric patients with PSC-IBD^26,27,35-39^ | 7 | 234 | 24.6 | 11.6-37.6 | 93.7 |
| PSC-IBD patients with prior orthotopic liver transplantation^15,17,22,26,^  ^39-47,68-75,86,91^ | 23 | 993 | 35.8 | 23.0-48.6 | 97.3 |
| PSC-IBD patients undergoing colorectal surgery^17,28-30,32,46,48-54, 80,86,87^ | 16 | 919 | 19.6 | 12.1-27.1 | 92.5 |
| **Sensitivity Analyses** | | | | | |
| Full-text studies (excluding abstracts) examining infections in PSC-IBD^13-66,75,76,79,83, 86,87,91^ | 61 | 3391 | 25.8 | 15.6-36.0 | 99.3 |
| Case series excluded^13-39,42,44-59,61-63,65-74,76-81,83, 84,87,92,93^ | 68 | 5353 | 24.2 | 15.7-32.7 | 99.2 |
| Abstracts and case series excluded^13-39,42,44-59,61-63,65,66,76,79,83,87,93^ | 54 | 3332 | 24.7 | 14.1-35.3 | 99.4 |
| High risk of bias studies excluded^13,15,17,19,20,23,27, 28,30-32,35,42,44,47,48,50-52,57, 59,71,72,78,79,81^ | 26 | 1992 | 26.5 | 8.0-44.9 | 99.7 |
| Studies published prior to year 2000 excluded^13,15-20,23,25-49,51,52,54-59,63,64,67-81,83,86,87,91-92^ | 64 | 5153 | 25.3 | 16.5-34.1 | 99.3 |

**Supplementary Table 6: Risk of bias of case-control studies using the Newcastle-Ottawa scale**

| **STUDY** | **SELECTION** | | | | **COMPARABILITY** | **EXPOSURE** | | | **SCORE** |
| --- | --- | --- | --- | --- | --- | --- | --- | --- | --- |
|  | **Adequacy of case definition** | **Representative-ness of cases** | **Selection of controls** | **Definition of controls** | **Comparability of**  **cohorts on the basis**  **of the design or analysis** | **Ascertainment of exposure** | **Same method of ascertainment** | **Non-response rate** |  |
| Benavente-Chenhalls 2008 | * | * | * | * | ** | * | * | * | 9 |
| Block 2014 | * | * | X | * | X | * | * | X | 5 |
| Eaton 2015 | * | X | X | * | ** | * | * | * | 7 |
| Gorgun  2005 | * | * | * | * | X | * | * | * | 7 |
| Hanouneh  2012 | * | * | * | * | X | * | * | * | 7 |
| Kochhar  2015 | * | * | * | * | NR | * | * | * | 7 |
| Lascurain  2016 | * | * | * | * | ** | * | * | * | 9 |

*NR*: not relevant. X: no points awarded.

Asterisks indicate the star rating according to the Newcastle-Ottawa Scale for cohort studies. A study can be awarded a maximum of 4 stars for Selection, 2 stars for Comparability, and 3 stars for Outcome.

**Supplementary Table 7: Risk of bias of cohort studies using the Newcastle-Ottawa scale**

| **STUDY** | **SELECTION** | | | | **COMPARABILITY** | **OUTCOME** | | | **SCORE** |
| --- | --- | --- | --- | --- | --- | --- | --- | --- | --- |
|  | **Representativeness of the exposed cohort** | **Selection of the non-exposed cohort** | **Ascertain-ment of exposure** | **Demonstration that outcome of interest was not present at start of study** | **Comparability of cohorts on the basis of the design or analysis** | **Assessment of outcome** | **Length of follow-up sufficient for outcomes to occur** | **Adequacy of follow-up of cohorts** |  |
| Barnabas (2014) | X | X | X | * | NR | NR | NR | NR | 1 |
| Bastón Rey (2022) | * | X | * | * | X | * | * | * | 6 |
| Bhardwaj (2016) | * | * | * | * | X | * | X | X | 5 |
| Bramuzzo (2016) | * | * | * | * | ** | * | * | * | 9 |
| Cangemi 1989 | * | * | * | * | X | * | * | X | 6 |
| Caron (2019) | * | X | * | * | * | X | * | X | 5 |
| Catassi (2023) | * | * | * | * | X | * | * | * | 7 |
| Charatcha-  roenwitthaya (2008) | * | X | * | * | * | * | * | * | 7 |
| Christensen (2019) | * | NR | * | * | ** | X | X | X | 5 |
| Dahiya (2024) | * | * | * | * | X | * | * | X | 6 |
| Drastich (2013) | X | NR | * | * | NR | * | * | * | 5 |
| Dunleavy (2023) | * | NR | * | * | X | * | * | * | 6 |
| Fain (2017) | X | NR | * | * | X | * | * | * | 5 |
| Guerra (2019) | * | * | * | * | ** | * | * | * | 9 |
| Halvoet (2024) | * | NR | * | * | X | * | * | * | 6 |
| Hedin (2020) | * | NR | * | * | * | * | * | X | 6 |
| Hensel (2021) | * | NR | * | * | * | X | * | * | 6 |
| Ida (2022) | X | NR | X | * | NR | * | * | * | 4 |
| Indriolo (2012) | * | NR | * | * | NR | * | * | * | 6 |
| Indriolo (2013) | * | * | * | * | X | * | * | * | 7 |
| Irles-Depe (2020) | * | * | * | * | * | * | * | * | 8 |
| Karime (2024) | * | NR | * | * | X | * | * | * | 6 |
| Kartheuser (1996) | * | * | * | * | X | * | * | * | 7 |
| Khettry (2003) | * | NR | * | * | X | X | * | * | 5 |
| Kulkarni (2021) | * | NR | * | * | ** | * | X | X | 6 |
| Laborda (2020) | * | NR | * | * | X | * | * | * | 6 |
| Lawlor (2018) | * | NR | * | * | X | * | * | * | 6 |
| Lee (2023) | * | * | * | * | * | * | * | * | 8 |
| Lemoinne (2019) | * | * | * | * | ** | * | X | X | 7 |
| Lian (2012) | * | * | X | * | X | * | * | X | 5 |
| Lynch (2020) | * | NR | * | * | ** | * | X | * | 7 |
| Malhi (2024) | * | * | * | * | * | * | X | * | 7 |
| Martin (1990) | * | * | * | * | X | X | * | X | 5 |
| Maspero (2023) | * | NR | * | * | X | * | * | * | 6 |
| Mathis (2008) | * | NR | * | * | X | X | * | * | 5 |
| Mathis (2011) | * | NR | * | * | * | * | * | * | 7 |
| Medina-Morales (2022) | * | * | * | * | ** | * | X | * | 8 |
| Miki (1995) | * | * | * | * | X | * | X | X | 5 |
| Mohabbat (2012) | * | * | * | * | X | * | * | * | 7 |
| Mouchli (2018) | * | * | * | * | * | * | * | * | 8 |
| Mousa (2019) | * | NR | * | * | X | * | * | * | 6 |
| Navaneethan (2012) | * | * | * | * | ** | * | * | X | 8 |
| Nedelkopoulou (2018) | * | * | * | * | X | X | * | * | 6 |
| Obusez (2013) | * | * | * | * | * | * | * | * | 8 |
| Osiecki (2021) | * | * | * | * | X | X | * | * | 6 |
| Pavlides (2014) | * | * | * | * | * | * | * | * | 8 |
| Peverelle (2018) | * | NR | * | * | X | * | X | * | 5 |
| Peverelle (2020) | * | * | * | * | ** | * | * | * | 9 |
| Poritz (2003) | * | NR | * | * | X | X | X | X | 3 |
| Post (1994) | * | * | * | * | X | X | X | * | 5 |
| Quinn (2022) | * | * | * | * | ** | * | * | * | 9 |
| Rabinovitz (1990) | * | * | * | * | X | X | * | * | 6 |
| Rasmussen (1992) | * | * | * | * | X | X | * | * | 6 |
| Rosenblatt (2019) | * | * | * | * | ** | * | * | * | 9 |
| Rupp (2014) | * | * | * | * | ** | X | X | X | 6 |
| Sayed (2023) | * | * | * | * | * | * | * | * | 8 |
| Schnitzler (2015) | * | * | * | * | X | * | * | X | 6 |
| Schregel (2023) | * | NR | * | * | X | * | * | * | 6 |
| Steinhagen (2009) | X | X | * | * | NR | * | X | * | 4 |
| Tobias (1983) | * | * | * | * | X | X | X | X | 4 |
| Treeprasertsuk (2013) | * | * | * | * | * | * | * | X | 7 |
| Tse (2019) | * | X | * | * | ** | * | * | * | 8 |
| Vandevrie (2003) | * | * | * | * | X | X | * | * | 6 |
| Venkat (2014) | * | NR | * | * | X | * | * | * | 6 |
| Warren (1966) | * | * | X | * | X | X | * | * | 5 |
| Wasmuth (2010) | * | * | * | * | * | X | * | * | 7 |
| Weng (2022) | * | * | * | * | X | X | X | X | 4 |
| Wright (2017) | X | X | * | * | X | * | * | * | 5 |
| Ye (2011) | * | * | * | * | X | X | * | * | 6 |

*NR*: not relevant. X: no points awarded

Asterisks indicate the star rating according to the Newcastle-Ottawa Scale for cohort studies. A study can be awarded a maximum of 4 stars for Selection, 2 stars for Comparability, and 3 stars for Outcome.

**Supplementary Table 8: Risk of bias of randomized controlled trial using the Cochrane Risk of Bias 2 tool**

|  | **Random sequence generation (selection bias)** | **Allocation concealment (selection bias)** | **Blinding of participants and personnel (performance bias)** | **Blinding of outcome assessment (detection bias)** | **Incomplete outcome data (attrition bias)** | **Selective reporting (reporting bias)** | **Other bias** | **Score** |
| --- | --- | --- | --- | --- | --- | --- | --- | --- |
| **Sandborn 1993** | ⊕ | ⊕ | ⊕ | ⊕ | ⊕ | ⊕ | ⊕ | 7 |

Risk of bias assessment for randomized controlled trials is based on the Cochrane Risk of Bias 2 tool. ⊕ indicates that the study has met the domain criterion, while an empty cell indicates that it is unclear whether the domain criterion has been met
